# Concerted suppression of all starch branching enzyme genes in barley produces amylose-only starch granules

**DOI:** 10.1186/1471-2229-12-223

**Published:** 2012-11-21

**Authors:** Massimiliano Carciofi, Andreas Blennow, Susanne L Jensen, Shahnoor S Shaik, Anette Henriksen, Alain Buléon, Preben B Holm, Kim H Hebelstrup

**Affiliations:** 1 Department of Molecular Biology and Genetics, Aarhus University, Aarhus, Denmark; 2 Department of Plant Biology and Biotechnology, VKR Research Centre for Pro-Active Plants, Faculty of Life Sciences, University of Copenhagen, Frederiksberg, Denmark; 3 KMC, Herningvej 60, Brande, 7330, Denmark; 4 The Protein Chemistry Group, Carlsberg Laboratory, Copenhagen, Denmark; 5 UR1268 Biopolymeres Interactions Assemblages, INRA, Nantes, F-44300, France

**Keywords:** Amylose-only starch, Resistant starch, RNA interference, Starch branching enzymes, Starch bioengineering, Starch granules, Starch crystallinity, Barley

## Abstract

**Background:**

Starch is stored in higher plants as granules composed of semi-crystalline amylopectin and amorphous amylose. Starch granules provide energy for the plant during dark periods and for germination of seeds and tubers. Dietary starch is also a highly glycemic carbohydrate being degraded to glucose and rapidly absorbed in the small intestine. But a portion of dietary starch, termed “resistant starch” (RS) escapes digestion and reaches the large intestine, where it is fermented by colonic bacteria producing short chain fatty acids (SCFA) which are linked to several health benefits. The RS is preferentially derived from amylose, which can be increased by suppressing amylopectin synthesis by silencing of starch branching enzymes (SBEs). However all the previous works attempting the production of high RS crops resulted in only partly increased amylose-content and/or significant yield loss.

**Results:**

In this study we invented a new method for silencing of multiple genes. Using a chimeric RNAi hairpin we simultaneously suppressed all genes coding for starch branching enzymes (SBE I, SBE IIa, SBE IIb) in barley (*Hordeum vulgare L.*), resulting in production of amylose-only starch granules in the endosperm. This trait was segregating 3:1. Amylose-only starch granules were irregularly shaped and showed peculiar thermal properties and crystallinity. Transgenic lines retained high-yield possibly due to a pleiotropic upregualtion of other starch biosynthetic genes compensating the SBEs loss. For gelatinized starch, a very high content of RS (65 %) was observed, which is 2.2-fold higher than control (29%). The amylose-only grains germinated with same frequency as control grains. However, initial growth was delayed in young plants.

**Conclusions:**

This is the first time that pure amylose has been generated with high yield in a living organism. This was achieved by a new method of simultaneous suppression of the entire complement of genes encoding starch branching enzymes. We demonstrate that amylopectin is not essential for starch granule crystallinity and integrity. However the slower initial growth of shoots from amylose-only grains may be due to an important physiological role played by amylopectin ordered crystallinity for rapid starch remobilization explaining the broad conservation in the plant kingdom of the amylopectin structure.

## Background

Starch, a polysaccharide composed of glucose molecules, is a common constituent of higher plants and can be found in all the organs being the major forms in which carbohydrates are stored [1,2]. Biosynthesis and accumulation of starch takes place into two different forms of plastids, chloroplasts and amyloplasts, depending on the anatomical site. Starch produced in chloroplasts is called transient starch and is a primary product of photosynthesis, along with sucrose. Transient starch synthesised during daytime is degraded during the following night, providing a continued supply of sugars to sustain metabolism throughout the night and for export to sink organs [3]. Whereas synthesis of storage starch, in plastids, takes place in storage organs such as tubers, roots and cereal grains. Storage starch in cereal grains is a long term carbon store for the next generation where it is used as a source of energy during periods of dormancy and re-growth [4,5].

Glucose moieties in starch form two structural arrangements called amylose and amylopectin. Amylose is a linear, or slightly branched molecule in which the glucose units are joined end-to-end by α-1,4 linkages and typically represents about 25% of the starch granule [5,6]. Amylopectin, the most abundant component of starch, is a much larger branched molecule containing a backbone of glucose residues linked through α-1,4 linkages with around 5% of α-1,6 glycosidic bonds [7]. These two molecules are packed together in insoluble granules into layers alternating between layers of semi-crystalline amylopectin and layers of amorphous amylose.

Starch is economically important. It is the major source of calories in food and feed worldwide. It is also a functional polymer with potential to provide environmental friendly biomaterials [8]. In recent years there has been increasing interests in the potential health effects of starch intake since easily digestible polysaccharides are considered responsible for a large part of severe health disorders such as obesity, cardiac disease and diabetes [9].

A rapid hydrolytic degradation of the bulk of dietary starch takes place in the lumen of the small intestine, making it a highly glycemic carbohydrate [10]. However, degradability of starch can vary considerably depending on origin, composition and physical state. In 1982 Englyst and coworkers identified a portion of dietary starch resistant to enzymatic hydrolysis escaping degradation in the stomach and the small intestine. This fraction was termed “resistant starch” (RS) [11] and further analysis revealed that RS reaches the large intestine almost undigested, where it is fermented by anaerobic gut bacteria [12]. The major metabolic products of this fermentation are short chain fatty acids (SCFA) mostly butyrate, acetate and propionate [13,14]. SCFA, especially butyrate, are associated with many health benefits being the preferred source of energy for colonocytes triggering increased colonic blood flow, oxygenation and muscular contraction [14,15]. Additional health promoting effects of RS include lumen acidification that is associated with growth inhibition of potentially pathogenic bacteria in favour of beneficial probiotic bacteria, stimulation of excretion and degradation of cytotoxic metabolite and increase in the absorption of Na^+^, K^+^, Ca^2+^ and Mg^2+^[14-17]. RS can also inhibit inflammatory responses [18], stimulate cell differentiation controlling mucosal proliferation [12,19], genetic repair mechanisms and prevention of colon cancer [20].

The proportion of amylose in starch has a direct positive correlation with RS content, reduced digestibility and lower glycaemic responses [21-25]. The exact nature and structure of RS are still complex and elusive, but the amylose component seems to decrease substrate accessibility to amylases mainly due to process stability and the rapid formation of very stable, double-helical, polysaccharide aggregates during a re-crystallisation process termed *retrogradation*[23,26].

*In planta* production of starches with high proportion of RS has caught considerable attention since such an approach can generate valuable enhanced health promoting qualities directly in the crop. Bioengineering has successfully provided increased amylose starches in wheat [21,27], rice [28,29], potato [30-33] and barley [34]. However, drastic yield penalty is observed [32] and a pure-amylose line has never been produced before, and it is therefore general consensus that biosynthesis of pure amylose cannot be achieved directly in plants. By silencing all genes for starch branching enzymes (SBE I, SBE IIa, SBE IIb) in barley with a single RNAi hairpin, we here demonstrate for the first time a concerted suppression of all SBE genes, and show that this results in high yield production of pure amylose starch directly in cereal grains. This plant system provides a strategy for the production of a novel pure functionalized starch composition with biomaterial and health benefits directly in cereal grains. Moreover, being the first true counterpart of the “waxy” or amylopectin-only starch types, the amylose-only barley represents a valuable model plant for fully understanding the full range of starch structural parameters from 0% to 100% amylose and its physicochemical properties [6].

## Results

### Transformation of transgenic plants

**Figure 1:**
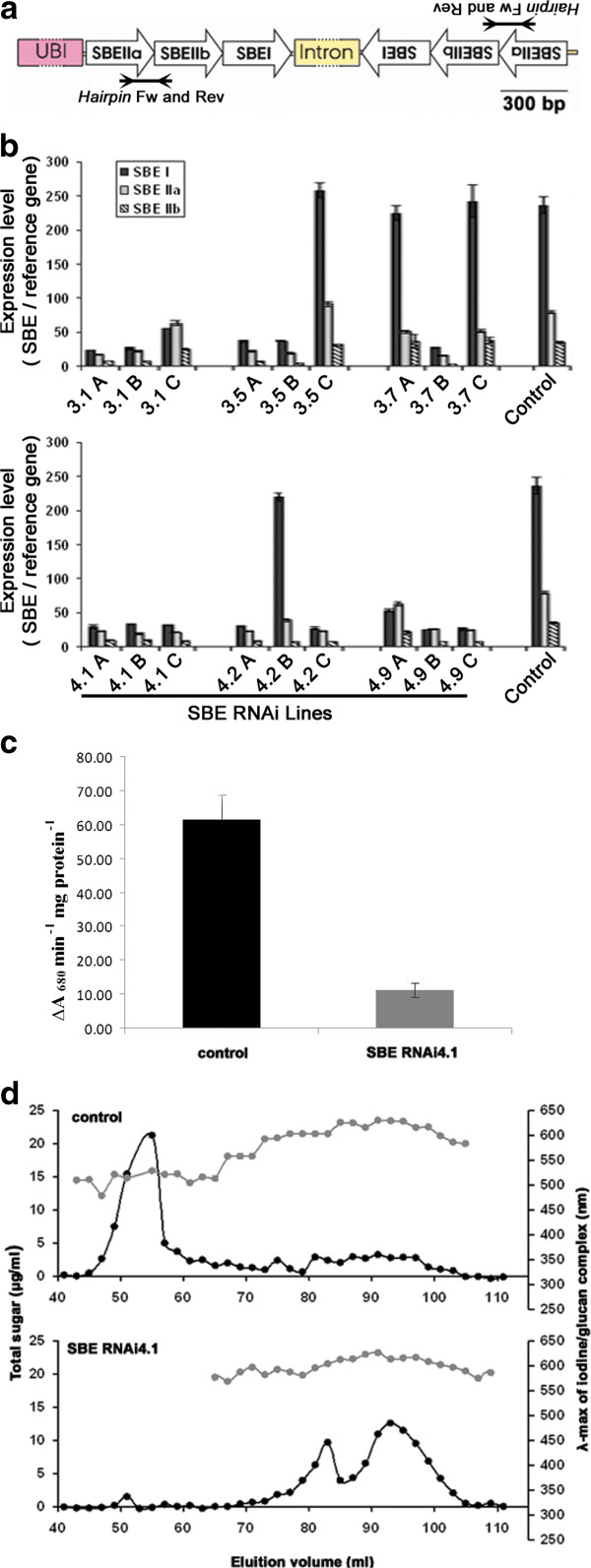
**Generation and identification of amylose-only barley.** (**a**) Chimeric RNAi hairpin construct simultaneously targeting the three different SBE genes SBEI, SBEIIa and SBEIIb. Expression was driven by the maize Ubiquitin promoter. Promoter and intron are not drawn to scale. The actual length of the intron is 1290 bp. The amplification product of the primer set *Hairpin* Fw and *Hairpin* Rev, which specifically recognizes the hairpin construct is indicated (**b**) Relative gene expression levels of the three SBEs isoforms (SBEI, SBEIIa and SBEIIb) assessed by RT qPCR in three individual grains, A, B and C at (20DAP) each of control and transgenic T_1_ lines (three technical replicates each). SE bars are indicated. (**c**) SBE enzyme activity in developing endosperm of SBE RNAi 4.1 and control grains, based on the average value of 3 experiments. Bars indicate standard error (**d**) Size exclusion chromatography (SEC) profile of starch from control and SBE RNAi4.1 lines. Black lines show the elution profile determined measuring the total sugar content of each fraction. λ-max absorbance of the α-glucan-iodine complex in each fraction is indicated by grey dots.

We intended to silence all three genes of the family of starch branching enzymes in barley by RNAi. Earlier studies suggests that silencing of an entire family of genes may be done either by targeting a highly conserved region, if one exists in all members of the gene family, or alternatively by designing a chimeric single construct with a sequence of multiple specific targets [35]. We did not find enough homology among those genes to design a single target sequence, and therefore a chimeric construct with three elements targeting each of the three SBE genes was constructed (Figure 1a). There was very little homology among the three different target sequences (Additional file 1). Transgenic barley lines of the cultivar Golden Promise were generated by *Agrobacterium*-mediated transformation.

### RT-qPCR screening and propagation of transgenic barley lines

11 (T_0_) independent transgenic plants were generated. Insertion of the selection marker gene (*Hpt*) was confirmed by PCR (gDNA from leaves). Expression of hairpin construct was detected in 6 of the lines (cDNA from developing endosperm 20 DAP) by RT qPCR (data not shown). The two lines SBE RNAi3 and SBE RNAi4, which showed the highest transcription levels of hairpin construct were selected and ten grains per line were propagated to generate T_1_ lines.

Transcription levels of the *Hpt* selection marker, hairpin constructs expression (cDNA from leaves, Additional file 2), SBE isoforms I and IIa (cDNA from leaves) (data not shown) and SBE isoforms I, IIa, IIb (cDNA from the endosperm of three different developing grains at 20 DAP called A, B and C, Figure 1b) were analyzed using RT qPCR in T_1_ generation lines. Six plants, SBE RNAi3.1, SBE RNAi3.5, SBE RNAi3.7, SBE RNAi4.1, SBE RNAi4.2, SBE RNAi4.9, were found positive for transgenes expression and SBE downregulation. At the level of T_1_ plants both homozygous and hemizygous plants will be usually be present. Therefore among the three offspring grains (T_2_) called A, B and C for each of the plants there will be some that are not transgenic. In agreement with this we observed both grains with SBE silencing and grains without gene silencing from some of the plants. Two plants, SBE RNAi4.1 and SBE RNAi 4.9, showing the highest level of gene expresssion suppression, were selected. The transcript levels of the three SBE isoforms detected in SBE RNAi4.1 endosperms as compared to control were 13% for *SBEI*, 27% for *SBEIIa* and 26% for *SBEIIb*. For the SBE RNAi4.9 line the values were 15% for *SBEI*, 48% for *SBEIIa* and 36% for *SBEIIb* (average values of three biological replicates). SBE RNAi4.1 and control plants were further propagated (T_2_) in greenhouse and in semifield trials.

### SBE activity assay

We extracted enzymes and measured starch branching activity in the endosperm of developing grains (15–20 DAP) from SBE RNAi4.1 (T_3_) and control plants grown in the greenhouse. With similarity to the reduction in gene expression (Figure 1b), we found that starch branching activity in the suppressed line SBE RNAi.4.1 was reduced by 82% as compared to the control grains (Figure 1c).

### Molecular size distribution analysis

Amylose concentrations and molecular size distribution were analysed by a combined size exclusion chromatography (SEC) and iodine complexation staining approach (Figure 1d). The chromatographic profile of control starch showed the major amylopectin peak eluting first with a maximum absorbance for the iodine starch-complex (λ-max) between 500 and 550 nm and a wide amylose fraction eluting later with characteristic λ-max between 580 and 630 nm. These values are characteristic for amylose and amylopectin respectively, and the amount of amylopectin can therefore be determined from the SEC from the area of the elution curve of samples with λ-max below 550 nm, whereas amylose content is determined from the area of the curve of samples with λ-max above 580 nm [36]. Using this method we found that the amylose/amylopectin ratio for the control starch was 29.9%/70.1%. In contrast the SBE RNAi 4.1 starch showed a double peak eluting almost all in fractions with λ-max above 580 nm, suggesting that these fractions are all amylose. Traces of amylopectin, found in a single fraction at 51 ml corresponds to an amylose/amylopectin ratio of 99.1%/0.9% (mean average of four technical replicates).

### Thermal, swelling and solubility characteristics

**Figure 2:**
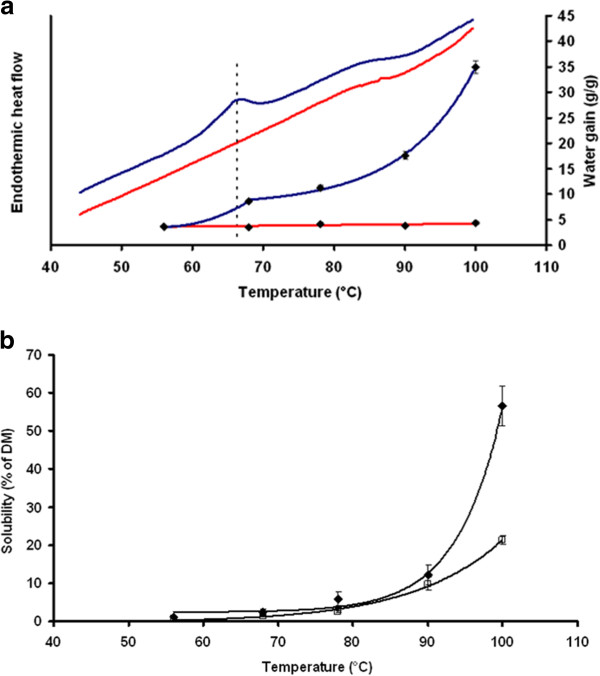
**Solubility.** (**a**) Thermal properties and swelling power of starch from SBE RNAi4.1 and control lines. Upper lines (red, SBE RNAi4.1; blue, control) show the endothermic heat flow and lower dots and lines show the water gain of starch. Vertical line indicates melting of amylopectin. The swelling power of starch after gelatinization at 100°C is reported as the ratio of water gain of the swollen starch pellet compared to starch dry matter. (**b**) Solubility (1% starch granule suspensions in water) of control (filled squares) and amylose-only (open squares) starch as a function of temperature. The solubility is reported as the ratio of total carbohydrate in the supernatant to total starch.

Thermal properties were analysed with DSC for the control starch and for starch extracted from the SBE RNAi 4.1 barley line (Figure 2a). Control starch had a peak gelatinization temperature at 66°C typical for amylopectin melting. No melting endotherm was detected in pure-amylose starch confirming virtual lack of normal amylopectin. In some samples a minor and very broad transition could be noted but this could be neither integrated nor quantified. An endotherm at approx. 95°C was seen for the SBE RNAi4.1 starch when melting at higher temperatures (data not shown). Peak melting temperature and melting enthalpy, ΔH, of amylopectin in control starch is shown in Additional file 3.

Swelling power trials demonstrated that the control starch started to swell at 68°C correlating with the DSC point of gelatinization at 66°C (Figure 2a). The swelling of the control starch increased up to 100°C at which point this starch had swollen by 35 g water per gram of starch. While starch from SBE RNAi4.1 did not swell and remained stable at below 4 g water per gram of starch in the temperature interval tested. Both starch types showed similar increased solubility, to approximately 70–80°C, reported as the ratio of total carbohydrate in the supernatant to total starch (Figure 2). After this point solubility of control starch dramatically increased up to approximately 60%, while solubility of amylose granules only increased to 20%. Hence, the amylose-only starch has a dramatically decreased capacity for thermal hydration, swelling and solubilisation.

### Barley grain internal morphology

**Figure 3:**
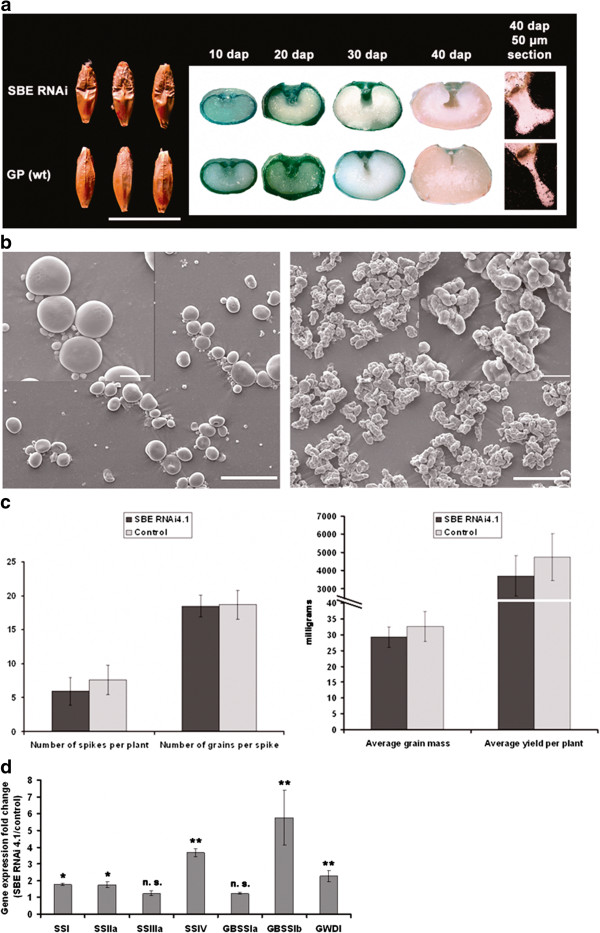
**SBE silencing affected grain shape, starch granule morphology, plants yield and expressions of other starch biosynthetic genes.** (**a**) Morphology, median cross sections and thin sections (50 μm) of representative SBE RNAi and control grains. (**b**) Scanning electron microscopy (SEM) pictures of control (left) and SBE RNAi4.1 (right) starch granules. Scale bars represent 50 μm and 10 μm in the lower and higher magnifications respectively. (**c**) Semi-field trial yield calculations, from left to right: average number of spikes per plants, average number of grains per spike, average grain mass and average yield per plant for SBE RNAi4.1 (dark grey) and control lines (light grey). Average yield per plant is expressed as total grain mass in milligram and SD bars are indicated. (**d**) Quantitative gene expressions level measured by RT qPCR of starch biosynthetic enzymes: starch synthases (*SSI*, *SSIIa*, *SSIIIa*, *SSIV*, *GBSSIa*, *GBSSIb*) and glucan water dikinase 1 (*GWDI*). Data are expressed in gene expression fold change between control and SBE RNAi4.1 lines (3 biological and 9 technical replicates per gene). SE bars are indicated. Genes with significant up-regulation in the SBE RNAi 4.1 line as compared to the control line are marked with * (P < 0.05) or ** (P < 0.01).

Transgenic grains had a characteristic wrinkled shape (Figure 3a). To check whether the grains internal morphology was affected a study with stereo binoculars and light microscopy was conducted on grains median and cross sections. SBE RNAi grains displayed expanded endosperm cavities with a bilobated shape on the side bordering the endospermal transfer cells (Figure 3a).

### Segregation ratio of the wrinkled phenotype

All the grains of each positive T_1_ plant were collected and the ratios of wrinkled/wild type (wt) grains were assessed for each plant (Table 1). The wrinkled trait was segregating in a classic 3:1 Mendelian ratio. To analyse if this segregation of phenotype is linked to segregation of the transgene, we isolated genomic DNA from seedlings germinated from 15 T_1_ grains (SBE RNAi 4.9) and tested for insertion of transgene by PCR using the primers *Hairpin* Fw and Rev (Figure 1a), which specifically recognizes the hairpin construct (Additional file 4 – upper panel). PCR amplification of the gene GAPDH was used as a positive control (Additional file 4 – lower panel). Ten of the grains had a wrinkled phenotype and these also contained the transgene hairpin, whereas 5 of the grains had a wild type phenotype and similarly the transgene hairpin was not detected in those, This indicates that the transgene hairpin segregates with the wrinkled phenotype.

**Table 1:** **Segregation ratio of wrinkled phenotype in T**_**1**_**generation SBE RNAi lines**

**SBE RNAi line (T**_**1**_**)**	**Total grains (T**_**2**_**)**	**Wrinkled grains**	**Wild type grains**	**Segregation ratio**
3.1	140	107	33	3.2:1
3.5	114	83	31	2.7:1
3.7	107	76	31	2.4:1
4.1	50	50	0	-
4.2	101	76	25	3.0:1
4.9	130	98	32	3.0:1

### Grain chemical composition

Whole flour from milled second generation (T_1_) SBE RNAi4.1, SBE RNAi4.9 and control mature grains was used for β-glucan analysis. β-glucan contents in SBE RNAi4.1 and SBE RNAi4.9 wrinkled grains were 22% and 33% higher than the content of control grains (Additional file 5).

The starch content in control grains, 52.8% w/w ± 2.3 S.D, as compared to starch content measured in SBE RNAi4.1 grains, 47.2% w/w ± 1.4 S.D, demonstrates that starch accumulation in transgenic grains was at level comparable to wild type barley. No difference in phosphate content between only-amylose and control starch was found.

### Starch granule structure

**Figure 4:**
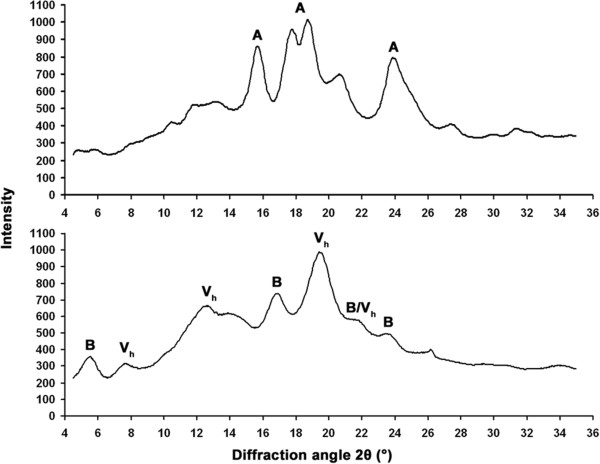
**Powder X-ray diffraction analysis (XRD).** X-ray powder diffractograms of control barley starch granules (top) and the amylose-only starch granules from the SBE RNAi4.1 line (bottom). The diffraction peaks at 2θ typical for A-type, B-type and V_h_-type crystalline polymorphs are indicated.

The purified starch was used for polarization light microscopy (Additional file 6), scanning electron microscopy (SEM) (Figure 3b) and powder X-ray diffraction analysis (XRD) (Figure 4). The granules did not show any birefringence indicating no main molecular direction of the glucose-chains (Additional file 6). As visualized with SEM large disc-shaped A-type and spherical small B-type granules were present in control starch. Multi-lobed, often elongated, rough and globose-shaped granules with no regular size distribution and a very rough surface morphology were prominent in amylose-only starch (Figure 3b). The multi-lobed morphology of amylose-only granules may be explained by abnormal multiple initiations followed by fusions of small granules. The same characteristic morphology was observed in granules prepared from grains of T_1_, T_2_ and T_3_ generations.

The diffraction peaks detected with XRD at 2θ for the control starch were typical for A-type (15.6°, 17.8°, 18.7°, 23.9°) crystalline polymorphs with approximately 20% crystallinity. For the SBE RNAi4.1 starch a combination of B-type (55% contribution at 5.6°, 16.7°, 21.7°, 23.6°, 26.1°) and V_h_-type (45% contribution at 7.6°, 12.6°, 19.5°, 21.7°) crystalline polymorphs with totally 25% crystallinity of the starch and no trace of the original A-type polymorph was found.

### Yield

Average number of spikes per plant was calculated from all semifield plants (Figure 3c Additional file 6a). A sampling was made selecting 5 random plants per pot (for a total of 25 individual plants per line). All the grains were counted in all spikes of these plants and the average number of grains per spike was calculated (Figure 3c Additional file 6b). Grains from each plant were collected and weighed to determine the average mass of the grains (Figure 3c, Additional file 7c). And finally the average yield per plant was estimated (Figure 3c, Additional file 7d). Pearson correlation coefficients (r) were calculated to assess the strength of the linear dependence among the yield components (Additional file 7e), demonstrating that yield loss in SBE RNAi plants was mainly due to a lower number of spikes per plant, and to a lesser extent also due to a lower mass of single grains. This was seen as a decrease of 21.7% in the average number of spikes per plant and 10.3% in the average mass of the single grains in the amylose-only line SBE RNAi4.1 compared to control line. No difference was present in the average number of grains per spike among the two lines.

### Plant height

**Figure 5:**
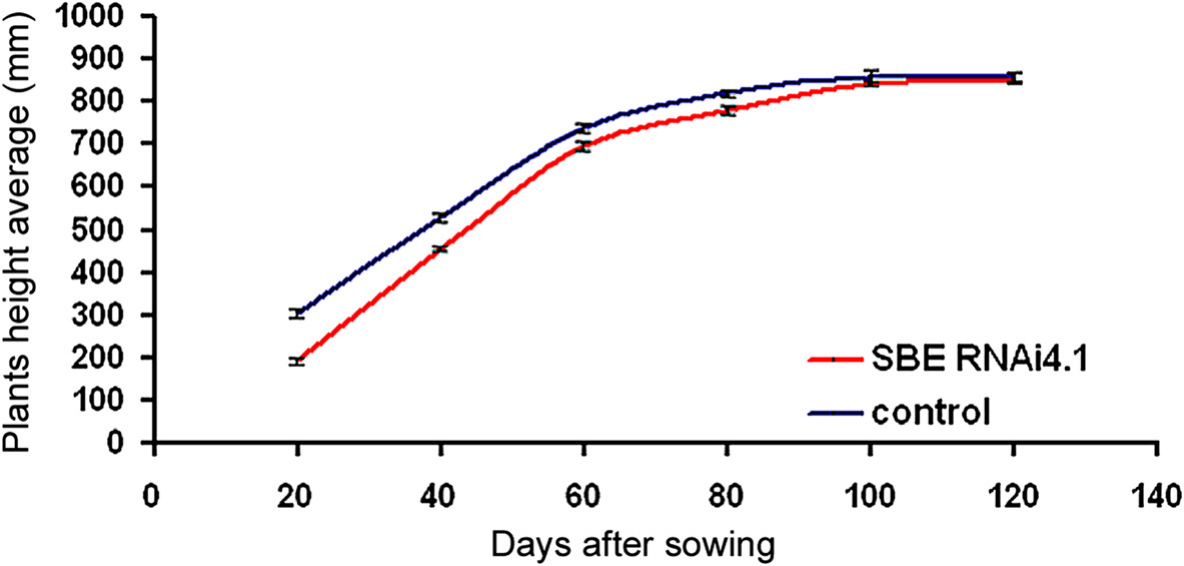
**Plant height.** Height average of plants of control (blue) and SBE RNAi4.1 (red) T_2_ lines. Plant heights (22 individuals for SBE RNAi 4.1 and 15 individual for control) were measured at 20, 40, 60, 80, 100, 120 days after sowing. SE is indicated.

Heights of T_2_ plants from control and SBE RNAi4.1 lines growing in greenhouse were measured at 20, 40, 60, 80, 100 and 120 days after sowing (Figure 5). Measurements showed that 20 days after sowing the SBE RNAi shoots were 37% shorter than control shoots, however this gap decreased gradually during plants growth, and was absent at full maturity at 120 days after sowing. These data indicate an initial slower growth of SBE RNAi plants.

### Germination frequency and starch remobilization during germination

No difference was found in the germination frequency of SBE RNAi4.1 and control lines grains sown in semifield trial (Additional file 8).

*In vitro* germination tests were conducted in destilled water in the dark to examine how biomass is remobilized into the new organs emerging at germination: radicle and coleoptie. After 12 days 85.4% of grain mass was lost in control grains, whereas only 66.8% of grain mass was lost in SBE RNAi4.1 grains (Table 2). Similarly, mass distribution into grain, radicle and coleoptile at day 12 demonstrated a lower biomass remobilization from grains into the emerged organs in the SBE RNAi4.1 grains compared with control grains, suggestion a slower degradation of the endosperm starch in the SBE RNAi4.1 grains (Table 3).

**Table 2:** ***In vitro *germination in dark: percentage of dry mass mobilization from grains from SBE RNAi 4.1 and control lines, 12 days after germination (average values of 10 grains from each line)**

**Line**	**Dry grain mass (day 0) ± SD (mg)**	**Dry grain mass (day 12) ± SD (mg)**	**Grains mass loss (%)**
SBE RNAi4.1	29 ± 2.7	9.6 ± 1.3	66.8%
Control	35.5 ± 8.5	5.2 ± 1.2	85.4%

**Table 3:** ***In vitro *germination in dark: relative dry mass distribution for grain, radicle and coleoptile from SBE RNAi 4.1 and control lines 12 days after germination (average values of 10 grains from each line)**

**Line**	**Dry masses (day 12) ± SD (mg)**			**Relative shoots mass composition (day 12)%**		
	**Grain**	**Radicle**	**Coleoptile**	**Grain**	**Radicle**	**Coleoptile**
SBE RNAi4.1	9.6 ± 1.3	3.4 ± 0.6	5.4 ± 2.3	79.3%	8%	12.7%
Control	5.2 ± 1.2	5.5 ± 1.7	8.5 ± 2.3	51.3%	19.1%	29.6%

### Pleiotropic effects of SBE RNAi silencing on gene expression of starch synthesis genes

Possible pleiotropic effects of the SBE RNAi silencing on the transcription levels of other starch biosynthetic genes were investigated by RT-qPCR for starch synthase (SS) *SSI*, *SSIIa*, *SSIIIa*, *SSIV*, granule bound starch synthase (GBSS) *GBSSIa*, *GBSSIb*, glucan water dikinase (GWD) *GWDI* in T_2_. RT-qPCR was performed in 20 DAP developing endosperms of SBE RNAi4.1 and control lines. Genes with significant up-regulation in the SBE RNAi 4.1 line as compared to the control line were *SSI* (1.7-fold), *SSIIa* (1.7-fold) and *GWDI* (2.2-fold). The most evident up-regulations were found for *GBSSIb* (5.7-fold) and *SSIV* (3.6-fold). All of these were mean values of 3 biological and 9 technical replicates (Figure 3d).

### *In vitro* starch degradation analysis and determination of resistant starch

**Figure 6:**
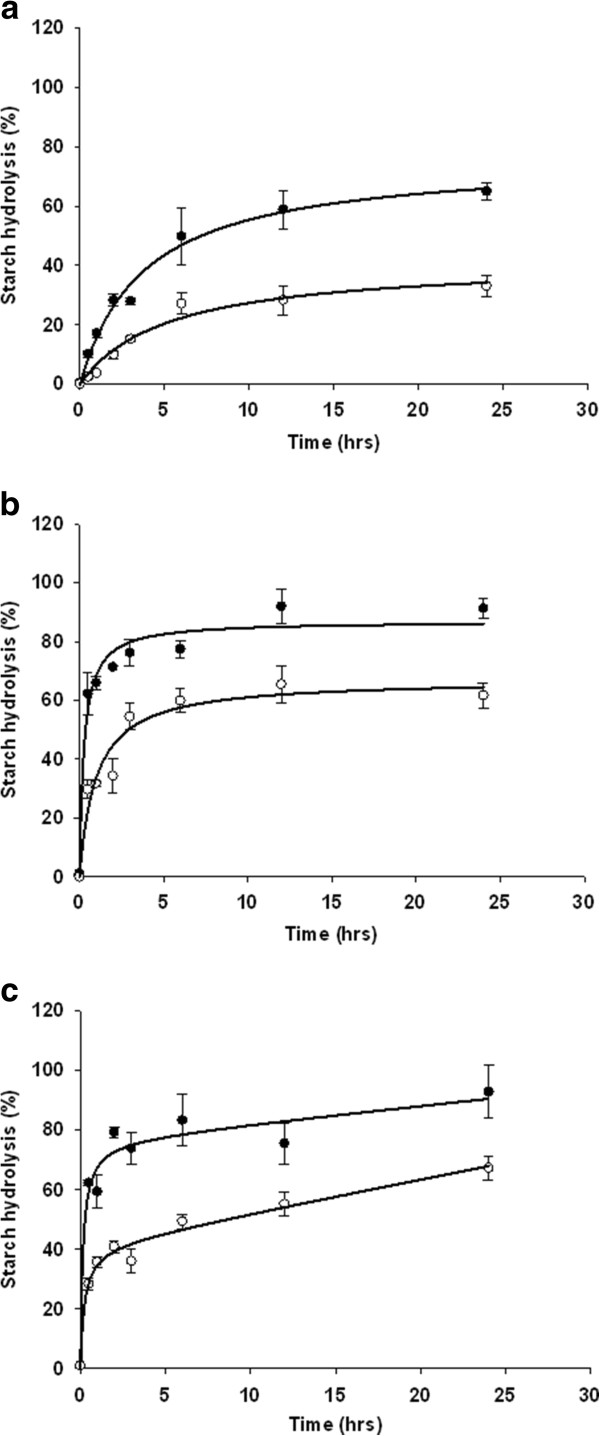
**Concerted *in vitro *enzymatic degradation of starch by pancreatic alpha amylase and glucoamylase.** Filled dots and empty dots indicate control starch granules and amylose granules respectively. SD bars are indicated. (**a**) Native starch granules. (**b**) Gelatinized starch. (**c**) Gelatinized and retrograded (re-crystallised) starch.

*In vitro* degradation by pancreatic α-amylase and glucoamylase was employed to simulate the effects of small intestine hydrolysis and subsequent glycemic response of the engineered starch [37]. The assay was carried out for both native starch, gelatinized starch and retrograded starches, and in all three situations SBE RNAi4.1 starch was much more resistant to degradation than normal barley Golden Promise control starch (Figure 6). For the native and the gelatinized starches (Figure 6A & B) data points were fitted using the Sigma plot package (Systat Software Inc) to a two parameters model: f =ax/(b+x), where “x” is the time and f the extent of degradation; “a” can be regarded as maximum asymptote and “b” can be regarded as the time to reach half of the maximum (Table 4). Maximum asymptote “a” was lower for SBE RNAi4.1 than for control in both native and retrograde starch, whereas the time to reach 50% of maximum enzymatic hydrolysis “b” was higher for SBE RNAi4.1 than for control in both native and retrograde starch. This demonstrates higher resistance to enzymatic degradation in the SBE RNAi4.1 starch than in the control starch. Retrogradation from gelatinized starch (re-crystallisation for 36 h at 4°C) introduced a slow additional linear degradation parameter in the degradation curve, so that the data was better fitted to a three parameters model: f=ax/(b+x)+cx, where “c” is the linear component, which was 0.59%/hour in the control and 1.1%/hour for the amylose-only starch (Table 4).

**Table 4:** **Parameters derived from non-linear fits (Figure**6**) for enzymatic hydrolysis of control and SBE RNAi4.1 amylose-only starch**

**Sample**	**a (%)**	**b (hrs)**	**c (%/hrs)**	**Regression**
Control				
*native granules*	76.1	3.8	na	0.9933
SBE RNAi4.1				
*native granules*	41.5	5.2	na	0.9839
Control				
*gelatinized*	86.9	0.27	na	0.9834
SBE RNAi4.1				
*gelatinized*	67.1	1.0	na	0.9697
Control				
*retrograded*	76.7	0.15	0.59	0.9818
SBE RNAi4.1				
*retrograded*	41.4	0.24	1.1	0.9916

A 2 parameters fit, f=ax/(b+x), was used for the native and the gelatinized starches, and a 3 parameters fit, f=ax/(b+x)+cx, was used for the retrograded starch where “a” describes maximum asymptote, “b” time to reach half maximum asymptote and “c” is the linear component only valid for the retrograded sample.

na: not applicable.

Based on the degradation data, the content of rapidly digestable starch (RDS), slowly digestable starch (SDS) and resistant starch (RS) was calculated according to Englyst [37] definition (Table 5). The amount of RS in SBE RNA4.1 was higher than for control starch for both native, gelatinized and retrograde starch (Table 5). And similarly the amount of RDS was lower in SBE RNA4.1 than for control starch for both native, gelatinized and retrograde starch.

**Table 5:** **Content of RDS, SDS and RS in amylose-only SBE RNAi4.1 and control starch calculated from data in (Figure**6**) according to the Englyst**[37]**method**

**Starch type**	**Native starch**		**Gelatinized starch**		**Retrograded starch**	
	**Control**	**SBE RNAi4.1**	**Control**	**SBE RNAi4.1**	**Control**	**SBE RNAi4.1**
RDS (%)	6.8	1.6	41	20	42	13
SDS (%)	21	8.3	30	14	30	18
RS (%)	72	90	29	65	29	68

RDS: (% starch hydrolyzed within the first 20 min).

SDS: (% starch hydrolyzed within 120 min - % starch hydrolyzed within first 20 min).

RS: (100% - % starch hydrolyzed within 120min).

## Discussion

Starch branching enzymes (SBEs) play a pivotal role in amylopectin biosynthesis by catalysing chain transfer by cleavage of an α-1,4 linkage following a condensation of an α-1,6 linkage [38]. In cereal species, such as rice, maize, barley and wheat, there are three classes of starch branching enzymes (SBE I, SBE IIa and SBE IIb). Barley (cv. Golden Promise) was genetically transformed to increase the starch amylose content by blocking amylopectin biosynthesis through silencing of all SBE genes. A single, multifunctional DNA construct was designed with the intention to simultaneously target the expression of the three genes encoding isoforms of SBEs in barley by RNA interference (RNAi) (Figure 1a). We found that expression of all three SBE genes was simultaneously reduced in grains of transgenic plants (Figure 1b). There was very little sequence similarity among the target sequences of the three different SBE genes (Additional file 1), which suggest that the simultaneous silencing was not an effect of homologous inhibition to corresponding genes by one of the segments in the hairpin. This conclusion is in agreement with a similar approach in rice [35], where it was demonstrated that up to three members of a gene family could be specifically silenced by a single chimeric hairpin construct of non-homologous segments. In this work the authors also conclude that transitive RNA silencing where formation of siRNA extent beyond the target sequence does not occur for endogenous genes in rice. We did not study that in this work, however it is unlikely that this would have an effect in the SBE gene family because of low sequence similarity among SBEI, SBEIIa and SBEIIb. In line with the observed reduction of gene expression of the SBE genes we found that starch branching enzyme activity was reduced by 82% in the SBE RNAi4.1 line when compared with a wild type control line grown under similar conditions (Figure 1c). This shows that the reduction in gene expression similarly reduced the level of enzyme activity.

Using size exclusion chromatography (SEC), we found that the amylose fraction in control starch was 29.9% of the total starch. In contrast, the amylopectin constituted less than 1% of total starch in SBE RNAi4.1, where instead a major double peak characteristic for amylose was identified (Figure 1d). The λ-max for these fractions are all above 580 nm supporting that this starch fraction is amylose. Some residual starch branching enzyme activity was observed in the SBE RNAi4.1 line suggesting that biosynthesis of amylopectin requires a certain threshold (above 18%) of SBE activity. SBE activity below 18% of control is not capable of synthesising amylopectin and the possibility that the amylose deposited in the SBE RNAi4.1 line contains some degree of branching not detectable by the iodine staining cannot be excluded. However, the SBE RNAi4.1 line also had significant increased expression of some of the starch synthases (Figure 3d), increasing the capacity for biosynthesis of non-branched starch.

Amylopectin is a semi-crystalline material with distinct thermal characteristics [39]. The thermal and solubility properties of the starch of the amylose-only line was analyzed and compared to starch from control barley. The control starch had as expected a peak gelatinization endotherm typical for amylopectin when using differential scanning calorimetry (DSC) (Figure 2a and Additional file 3). This endotherm was completely absent in the SBE RNAi4.1 starch confirming the absence of normal amylopectin in this starch. The endotherm seen at approx. 95°C in SBE RNAi 4.1 starch (data not shown) is characteristic of the amylose Vh crystal polymorph and supports the presence of normal amylose. Together with the SEC data these results demonstrated that the phenotype was amylose-only with a characteristic molecular fingerprint of amylose. Silencing of two SBE genes *SBEIIa* and *SBEIIb* in barley increases amylose content to a certain degree (70%) [34]. Our data show that amylose-only barley can be obtained when simultaneous suppression of all of the three SBE genes is performed (Figure 1b). This effect underlines the important role played by SBEI in barley endosperm starch biosynthesis in contrast to the apparent non-functionality suggested for SBEI in *Arabidopsis* leaves [40] and wheat endosperm [41].

Swelling and solubilisation of starch in aqueous systems, e.g. during cooking, are crucial for efficient enzymatic starch digestion leading to glycemic response. Heating of granular starch in excess water disrupts the crystalline structure as an effect of breakage of the extensive hydrogen bonding network between water molecules and the hydroxyl groups of the starch. This causes granule swelling and gelatinization [42] and the branched amylopectin, but not amylose is primarily responsible for this effect [43]. In control line the start point of swelling coincided with melting of amylopectin at 66°C. Starch extracted from SBE RNAi4.1 did not show any visible swelling (Figure 2a).

Major suppression of enzymatic degradation rates and a major increase in the RS content fraction were found for the SBE RNAi4.1 starch as compared to control starch as evaluated by the Englyst method for determination of RS. The amount of RS in the amylose-only starch was 90%, 65% and 68% respectively for native, gelatinized and retrograded starches (Table 5). For comparison cooked banana and potato starches, which is considered very high in RS, do not exceed 30% RS. These data demonstrate important health-associated features of this novel all-native resistant starch. It also provides the last link to complete the compositional range of starch produced in the cell from 0% amylose, the so called waxy type starch [44], to 100% amylose to generate the entire range of amylose:amylopectin ratios in plants important for completing our understanding of starch bioengineering.

The transgenic grains had a characteristic wrinkled phenotype (Figure 3a) and the SBE RNAi4.1 endosperm cavity appeared elongated and enlarged. Interestingly, the wrinkled seed is a phenocopy of the pea phenotype *rugosus* described by Gregor Mendel in his study on the laws of inheritance published in 1865 [45-47], which is also due to a loss-of-function in SBE activity [45]. The easily recognizable phenotype allowed us to score segregation (Table 1). The phenotype segregated 3:1. The fact that the SBE RNAi construct permits simultaneous targeting of three independent SBE genes, is of particular practical importance in breeding. That is because segregation in a single locus is practically more feasible as compared to the traditional alternative of differential suppression by independent RNAi constructs targeting each of the SBE genes [34] or crossing of multiple individual SBE loss-of-function genes, which each segregates independently. The strategy has been presented previously by [35]. However this is to our knowledge the first time that the method has been applied in a situation where silencing of multiple independently segregating genes is necessary for achieving a particular biosynthetic product, which in our case is amylose-only starch. For higher plants this is especially important where many metabolic pathways are highly redundant due to presence of isoenzymes and gene families in metabolic networks [48,49] and single gene loss-of-function is therefore often phenotypically silent.

Increased amylose content in cereal grains has been demonstrated to be correlated with altered accumulation of others grain constituents like β-glucan and water content [50]_._ Similarly in SBE RNAi4.1 and SBE RNAi4.9 wrinkled grains the β-glucan content was significatively higher than in control barley grains (Additional file 5). Cereal grain β-glucan has been shown to be associated with important dietary health benefits [50].

Simultaneous suppression of the only two classes of starch branching enzymes, SBE I and SBE II present in dicotyledonous plants such as pea and potato using a single [32] or a sequential [30,33] round of transformation in potato led only to a partial suppression of the amylopectin content and a dramatic increase of starch phosphate. Here we found that SBE suppression in barley had no significant effects on the content of starch bound phosphate (data not shown). Hence, the starch generated in this study provides for the first time an amylose-only model with no effects on starch phosphate.

Starch granule morphology and structure were severely altered in the amylose-only chemotype (Figure 3b, Figure 4 and Additional file 6). Normal starch granule morphology and crystallinity arises from repeated amylopectin double-helical lamellae. The disordered morphology of the SBE RNAi granules therefore reflects the lack of ordered amylopectin and suggests the presence of abnormal multiple granule initiations typical for high amylose chemotypes [28]. These novel granules are expected to compose new combinations of crystal polymorphic packing. There are two main starch crystalline polymorphs: the A polymorph present in cereal seed starch and the B polymorph typically found in tuberous storage starch, transitory leaf starch and amylose-rich starch. A third single helical V_h_ polymorph is typical for amylose, especially in complexation with lipids, iodine or alcohols [51]. We found a shift from A-type in the control starch to a mixed B/Vh-type polymorph in the SBE RNAi line, typical for high-amylose starch [52]. Such starch is also associated with resistance to enzymatic hydrolysis and dietary fiber-like properties [52,53].

Yield and germination were investigated in the T_2_ generation of SBE RNAi 4.1 plants grown under semi-field conditions. An analysis of the individual components contributing to overall yield: spike number, grains per spike and grain weight showed that the yield penalty in the amylose-only barley is mainly due to fewer spikes per plant, and to a lesser extent lower grain mass. All together, the overall yield was 22% below that of control plants grown under identical conditions, which is much less dramatic as compared to other high-amylose systems [32]. Hence, the cereal system all-together has excellent potential for large-scale production of pure amylose. The wrinkled phenotype may indicate decreased starch content, however the starch content of the amylose-only grains was 47.2%, which is only slightly lower than in the control starch (52.8%).

No difference was found in the germination frequency of SBE RNAi4.1 and control grains (Additional file 8). However, SBE RNAi4.1 line plants exhibited slower growth of the young plantlet as compared to control but the difference disappeared throughout later development (Figure 5). We hypothesized that this effect indicates an impediment of endosperm starch remobilization, during early development when the plant is dependent on the starch as a carbon source. The *in vitro* dark germination showing less grain mass for the control than for the SBE RNAi4.1after germination confirmed this hypothesis. Less dry biomass had been redistributed to the coleoptile and radicle in the SBE RNAi4.1 grains (21%) compared with control grains (49%). This demonstrates the physiological importance of amylopectin in the structural ordering of carbohydrate to allow a more rapid energy remobilization.

The endosperm of developing SBE RNAi grains had increased expression levels of a number of key starch biosynthetic enzymes. The most prominent increases were found for *SSIV,* and for *GBSSIb* which was previously reported to be specifically expressed in pericarp rather than in endosperm [54]. In durum wheat where *SBEIIa* was silenced, a similar up-regulation of the genes encoding GBSSI, SSIII, Limit Dextrinase (*LD)* and Isoamylase 1 (ISAI) has been detected [27]. This general up-regulation of the starch synthases in cereals may explain how our amylose-only barley line can compensate starch synthesis, preventing severe yield loss as seen in e.g. high-amylose potato [32].

## Conclusions

An amylose-only starch was produced with high yield in the barley endosperm by implementing a new method of simultaneous suppression of the entire complement of genes encoding SBEs.

The severe yield penalty most often observed for these kind of enzyme suppressions is supposedly counteracted by pleiotropic stimulation of a number of starch biosynthetic enzymes and, generally, high yield storage of pure amylose in cereal seeds is of interest for industrial-scale production of this polysaccharide. The high amylose grains had a characteristic wrinkled phenotype, which segregated in a 3:1 ratio. The fact that amylose-only barley can be obtained only when suppressing the expression of all the SBE genes reveals the functional importance for all SBE genes. Finally, we demonstrated that amylose-only starch granules can be synthesized and deposited with very high proportion of V_h_ crystallites and that amylopectin is not essential for granule crystallinity and integrity. Such polysaccharides can have significant applications such as food additives to ensure improved health via large bowel fermentation of resistant starch.

## Methods

### Transgenic construct design and barley transformation

The tri-antiSBE sequence was synthesized artificially by Eurofins Medigenomix GmbH (Germany). For each SBE gene, 300 base pairs of coding sequences from the cDNA sequence were used as targets as shown in Additional file 1.

The 300 base-pair fragments were designed to be flanked by the restriction-enzyme target sites for SalI and XhoI. This resulted in a DNA fragment with the following syntax: SalI-SBEIIa-SBEIIb-SBEI-XhoI, which was cloned into the RNAi silencing vector pSTARGATE forming a chimeric triple RNAi hairpin construct.

To secure high expression, the construct was expressed under control of the maize Ubiquitin-2 constitutive promoter (2 kb) [55]. This fragment was subcloned into the vector pENTRY4 (Invitrogen) via SalI and XhoI. The resulting vector (pENTRY4-SBE-RNAi) was confirmed by direct sequencing, and recombined by LR clonase (Invitrogen) into the RNAi vector pSTARGATE by the following protocol (1 uL pSTARGATE vector (140 ng), 3 uL pENTR4-SBE-RNAi (240 ng), 4 uL LR Clonase, 4 uL TE Buffer pH 8.0, incubated at room-temperature for 18 hours. Sense and anti-sense sequences were separated by an intron (1.3 Kb). The resulting construct pSTARGATE-SBE-RNAi was cloned into *E. coli* DH5α as described by the manufacturer (Invitrogen). Plasmid DNA was purified by a ‘Fastplasmid Mini Kit’ as described by the manufacturer (5Prime, Germany) and analyzed by restriction enzyme digestion (BamHI or SmaI). Valid vectors were transfected to *Agrobacterium tumefaciens* (AGL0) and used for transformation of *Hordeum vulgare* var. Golden Promise. Transgenic barley transformation was performed as described [56].

### Quantitative real-time PCR

Genomic DNA and RNA analysis using quantitative real-time PCR (RT qPCR) was performed as described [56]. Relative quantification of expression was calculated using glyceraldehyde-3-phosphate dehydrogenase *GAPDH* as an internal control as described [57]. All the analyses were conducted in three technical replicates using the primers described in Additional file 9.

### Extraction from barley endosperm and SBE activity assay

Barley endosperms at 15–20 DAP were collected from 3 different plants (1 spike from each plant). Endosperms (5–7) were collected from each spike and pooled. The endosperms were homogenized in ice cold buffer (50 mM Tris–HCl with 10 mM EDTA, 10 mM DTT and a Protease inhibitor tablet, Roche company, pH-7.5). The homogenate was centrifuged at 15,000g for 15 min at 4°C. The supernatant was re-centrifuged at 15,000g for 10 min at 4°C to remove any traces of debris. Protein concentration was determined using a standard Bradford reagent (Sigma Life Science, Cat. No. B6916) and the samples were diluted to 1mg/mL protein and stored at −20°C until analysis. SBE activity was measured as the decrease in absorbance of the amylose-iodine complex after SBE catalyzed branching as described by [58]. Acarbose (1.4mM in the reaction volume) was used to inhibit interference with amylases. 10μl of extract was added in a microtiterplate well and 50μl amylose solution (0.5 mg/ml in 100 mM sodium phosphate buffer, pH 7) was added and mixed. Reactions were stopped at intervals by addition of 0.1 N HCl. The Lugol solution (5-fold diluted) was added and absorbance measured at 680 nm and activity expressed as ∆A680 min^−1^ mg protein^−1^.

### Starch extraction and purification

Starch was extracted and purified using a modified version of the protocol described [56]. To avoid possible effects on crystalline structure such as artificial formation of amylose/alcohols complexes upon isolation [51] the starch purification protocol [56] was modified by extraction with distilled water only and air dried at room temperature with no addition of alcohol or acetone. Due to segregation in the heterozygous SBE RNAi4.9 line starch from only transformed grains was purified and studied separately. Grains from the homozygous SBE RNAi4.1 line were pooled together and starch was purified. Starch from the barley variety Golden Promise grown under identical conditions was used as control.

### Iodine complexation analysis

Iodine colorimetric analysis was carried out as described [59].

### Size exclusion chromatography (SEC)

SEC was performed as described [60].

### Differential scanning calorimetry (DSC)

Samples were analyzed using a Perkin Elmer Diamond DSC instrument operated from 30 to 100°C at a scanning rate of 10°C per minute. The starch granules were analyzed in aqueous slurries consisting of of 3 mg starch granules and 12 μL 10 mM NaCl in technical duplicates. Perkin Elmer Pyris 7.0 software was used to determine the parameters peak temperature (TP) and enthalpy change (ΔH).

### Swelling power and solubility

Swelling power was determined using a modification of the method of Schoch (1964) [61]. For solubility determination a 1% (w/w) starch suspension of 1 mL ddH_2_O was placed in a pre-weighed centrifuge tube and vortexed. After heating for 20 min in a shaking thermomixer at 56, 68, 78, 90 or 100°C, the tube was cooled to 15°C and centrifuged at 15.000 ×g for 10 min. The supernatant was removed by siphoning, and the swollen, precipitated starch was weighed. The total carbohydrate content of the supernatant was determined in triplicate using a modified method of Dubois *et al.* (1956) [62]. Properly diluted sample or standard (30 μL) was transferred to a well in a microtiter plate along with 30 μL 5% phenol, concentrated H_2_SO_4_ (200 μL) was added and the absorbance read at 490 nm using glucose as standard.

### Binocular stereo and light microscopy of grains

Median cross sections were prepared using a scalpel and examined with a Wild MZ8 Leica stereo microscope. For light microscopy analysis 50 μm thin sections were cut from fresh frozen 40 dap grains using a HM 550 OM Cryostat microtome, stained with I2/KI and mounted on a Zeiss Axioplan 2 Imaging microscope.

### β-glucan content

β -glucan content was determined using the β-Glucan (Mixed Linkage) kit by Megazyme International Ltd. (Wicklow, Ireland) following the manufacturers protocol.

### Starch content

Starch content was determined using the ‘Total starch AOAC Method 996.11/AACC Method 76.13’ kit from Megazyme International Ltd. (Wicklow, Ireland) using the protocol recommended by the manufacturer for samples containing resistant starch.

### Phosphate content

Phosphate content was measured as described [56].

### Polarization light microscopy

Polarization light microscopy was performed as described [63].

### Scanning electron microscopy

Scanning electron microscopy was carried out as described [56].

### X-ray diffraction analysis (XRD)

Powder X-ray diffraction was performed following appropriate hydration as described [64]. XRD diagrams were recorded on a Bruker D8 Discover diffractometer (Wissembourg, France) and a Rigaku RU-H2R system. Relative crystallinity was determined after normalization of the diffractograms between 4 and 35° (2θ). A- and B-type re-crystallized amyloses and dry extruded potato starch were used as standards.

### Semifield trials

Semifield experiments were conducted in a locked out-door cage raising 50 plants in 5 100 L soil pots, 10 plants each pot. SBEs suppression and amylose-only starch composition in this consecutive generation was confirmed by RT qPCR, iodine complexation and DSC respectively (data not shown).

### Yield

Average number of spikes per plant was calculated from all plants. Average mass of the grains, average number of grains per spike and average yield per plant were calculated on a sampling. Statistical comparisons between the two lines (control and SBE RNAi4.1) were evaluated using a *t*-test (PROC ANOVA) with k-1 and n-k degrees of freedom, where k is 2 (control and SBE RNAi4.1) and n is the number of observations. A value of P < 0.05 was considered to indicate statistical significance.

### Plant height

Heights of T_2_ plants growing in greenhouse (22 individuals from line SBE RNAi4.1 and 15 individuals from control line) were measured at 20, 40, 60, 80, 100 and 120 days after sowing and the average height was calculated.

### Germination frequency and starch remobilization during germination

60 grains (T_2_) from lines SBE RNAi 4.1 and control each were sown in semifield trial and the germination ratios were calculated by inspection of germinated seedlings after 2 weeks.

Starch remobilization was conducted by incubating 10 grains each for SBE RNAi4.1 and control lines on filter paper soaked in water in germination boxes for 12 days. The boxes were placed at controlled temperature of 23°C in a dark growth chamber to avoid photosynthetic carbon fixation. At day 12 the germinated seedlings were collected. The coleoptile, the radicles and the grain were excised using a scalpel to be desiccated separately and determine distribution of dry mass into each of these organs and the dry mass loss from the grain. To calculate the initial dry mass of the grains per line, average water content of mature T_2_ grains was estimated by weighing 10 grains from SBE RNAi4.1 and 10 grains from control line before and after a desiccation treatment of 24 hours at 95°C in a ventilated oven.

### *In vitro* starch degradation analysis

*In vitro* starch degradation was analyzed by a modification of the Englyst method [37], using native raw starch granules, gelatinized starch granules (98°C, 12 min), and retrograded starch i.e. gelatinized starch re-crystallized for 36 h at 4°C. Starch samples (2% in 250 μl), were incubated in duplicates with 2U of each α-amylase from porcine pancreas (Sigma A3176) and amyloglucosidase (*A. niger*, Fluka 10113) in 20 mm sodium phosphate buffer with 6.7 mM sodium chloride (pH 6.0) at 37°C for 0, 0.5, 1, 2, 6, 12, 24 hours. Enzyme treatment was terminated by adding 30 μl 0.1 M HCl and 250 μl of 50% ethanol on ice and centrifuged (14,000 g, 5min) and the supernatant was collected. The amount of soluble reducing sugars was measured [62] and the rate of starch digestion was expressed as the % of glucose released from the added starch over the time period.

## Competing interests

The authors declare that they have no competing interests.

## Authors’ contributions

MC carried out transgenic plants regeneration, quantitative real-time PCR, starch extraction and purification, iodine complexation analysis, size exclusion chromatography, binocular stereo and light microscopy of grains, β-glucan content, starch content, phosphate content, scanning electron microscopy, yield assessment, plant height analysis, germination frequency test, starch remobilization analysis and wrote the manuscript. AB assisted in the writing of the manuscript, supervised, planned and coordinated the work on starch analysis and assisted in the size exclusion chromatography. SLJ carried out differential scanning calorimetry, swelling power and solubility analysis, prepared text and figures relative to these experiments and collaborated in the size exclusion chromatography and X-ray diffraction analysis. SSS carried out starch extraction and purification, SBE activity assay, polarization light microscopy, *in vitro* starch degradation analysis and prepared text and figures relative to these experiments. AH and AB carried out X-ray diffraction analysis. PBH assisted in the writing of the manuscript. KHH designed the transgenic construct, carried out the transgenic transformation, assisted with binocular stereo microscopy of grains and size exclusion chromatography, prepared text and figure relative to construct design and transgenic transformation, assisted in the writing of the manuscript and planned, supervised and coordinated the project. All authors read and approved the final manuscript.

## Supplementary Material

Additional file 1 **SBE target sequences.** Target sequence of SBEI, SBEIIa and SBEIIb for the chimeric SBE RNAi construct. Click here for file

Additional file 2 **Transgene expression analysis.** Relative expression levels of selection marker gene (*Hpt*) and transgenic hairpin construct analysed by RT qPCR in leaves of control and transgenic T_1_ lines (three technical replicates each). SE is indicated. Click here for file

Additional file 3 **Differential scanning calorimetry table.** Differential scanning calorimetry (DSC) in aqueous suspension of starch extracted from control and from SBE RNAi4.1 lines. ΔH: Change in enthalpy due to starch thermal dissolution in water. Click here for file

Additional file 4 **PCR of SBE RNAi 4.9 T**_**1**_**genomic DNA.** A PCR was performed using the primers *Hairpin* Fw and Rev (Figure 1a) to detect presence of the transgene hairpin in the genomic DNA of SBE RNAi 4.9 T_1_ grains. Primers for GAPDH was used as a positive control. The experiment was conducted in triplicate showing the same result. Click here for file

Additional file 5 **β-Glucan content. β-Glucan content in SBE RNAi4.1, SBE RNAi4.9 and control T**_**2**_**grains.** β-glucan content is reported in percent dry weight (average values of two biological and six technical replicates). Click here for file

Additional file 6 **Polarization microscopy.** Bright field microscopy image (left) and polarized microscopy image (right) of control starch granules (a, b) and amylose-only granules from line SBE RNAi4.1 (c, d). Click here for file

Additional file 7 **Yield.** (a) Average number of spikes per plant calculated in all plants from a semi-field trial (sample population: 50 plants each line). (b) Average number of grains per spike in 25 plants per line from a semi-field trial. (c) Average single grain mass calculated for 25 plants per line from a semi-field trial. (d) Average yield per plant in milligram of grain produced calculated in a sample of 25 plants per line from semi-field trial. (e) Correlation coefficients (r) and significance level as P values for the three yield components: average yield per plant, average number of spikes per plant and average grain mass. Click here for file

Additional file 8 **Germination frequency.** Germination frequency in soil of barley grains from SBE RNAi 4.1 and control lines. Click here for file

Additional file 9 **Primers.** Primers for RT qPCR. Click here for file
