# Supplementary material for: Concerted suppression of all starch branching enzyme genes in barley produces amylose-only starch granules
Source: BMC Plant Biol. 2012 Nov 21;12:223. doi: 10.1186/1471-2229-12-223 (PMC3537698; doi:10.1186/1471-2229-12-223)

| Gene | Accession number of sequence | 300 bp target sequence:  5’- 3’ |
| --- | --- | --- |
| SBEI | AY304541 | agcaagctctctcttcccgtgtctgcgccaagagacttcactatggcaacagctcaagatggtgccgacgaccttcccatatacgatctggatcccaagttcgccggcttcaaggatcacttcagttacaggatgaaaaagtaccgtgaccagaaacatctgattgacgaacacgagggaggccttgaagagttctctaaaggctatttgaagtttgggatcaacacagaaaatgatgcaactgtgtacagggaatgggcccctgcagcaaaggaagcacaggttattggtgacttcaactcgag |
| SBEIIa | AF064560 | atggcggaagtaaacatgacagggggggctgcagaaaaacttgaatcttcagaaccgactcagggtattgcggaaacaatcactgatggtgtaaccaaaggagttaaagaactagtcgttggggagaaaccgcaagttgtcccaaaaccaggagatgggcaaaaaatatacgagattgacccaacgctgaaagattttcggagccatcttgactaccgatacagcgaatacaagagaattcgtgctgctattgaccaacatgaaggtggattggaagttttttctcgtggttatgaaaag |
| SBEIIb | AF064561 | atggcggcgccggcgttcgcagtttccgcggcggggatcgcccggccatcggctcgtcgatccagcggggcagagccgagatcgctgctcttcggccgcaacaagggcacccgtttcccccgtgccgtcggcgtcggaggttctgggtggcgcgtggtcatgcgcgcgggcggcccgtccggggaggtgatgatccctgacggcggtagtggcggaagcggaacaccgccttccatcgagggttccgttcagttcgagtctgatgatctggaggttccattcatcgacgatgaaccaagc |

Alignments:


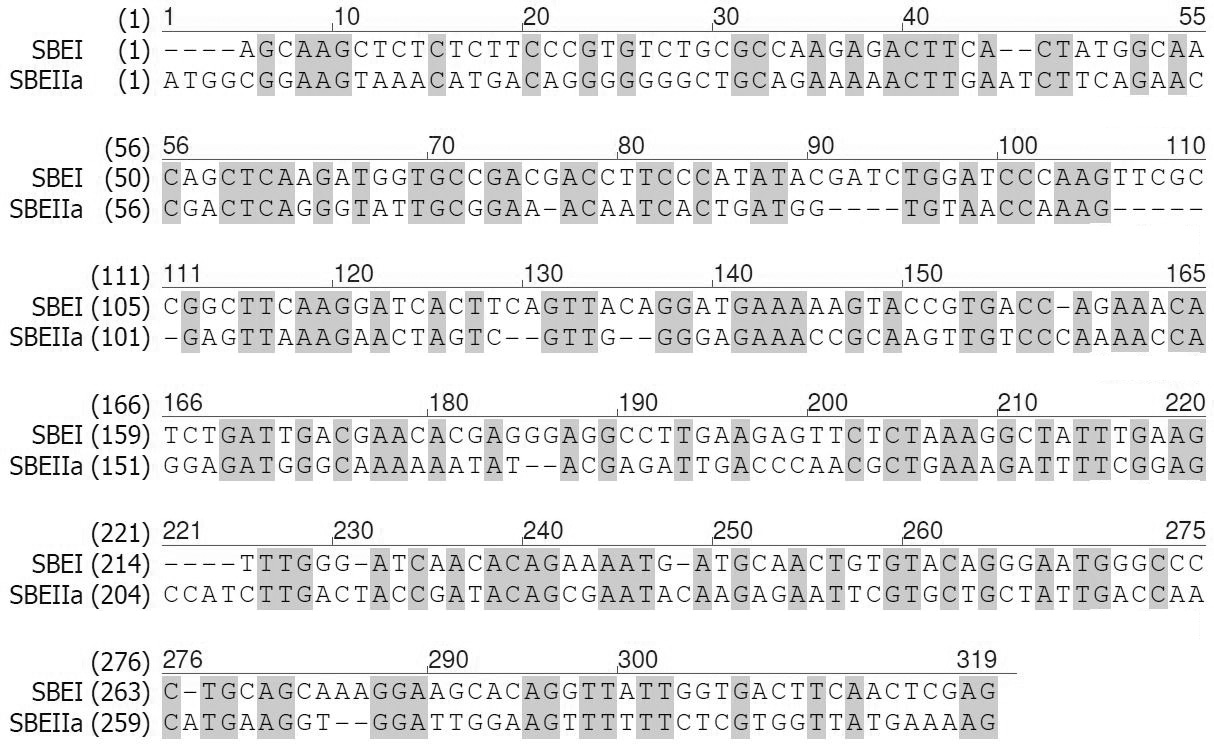


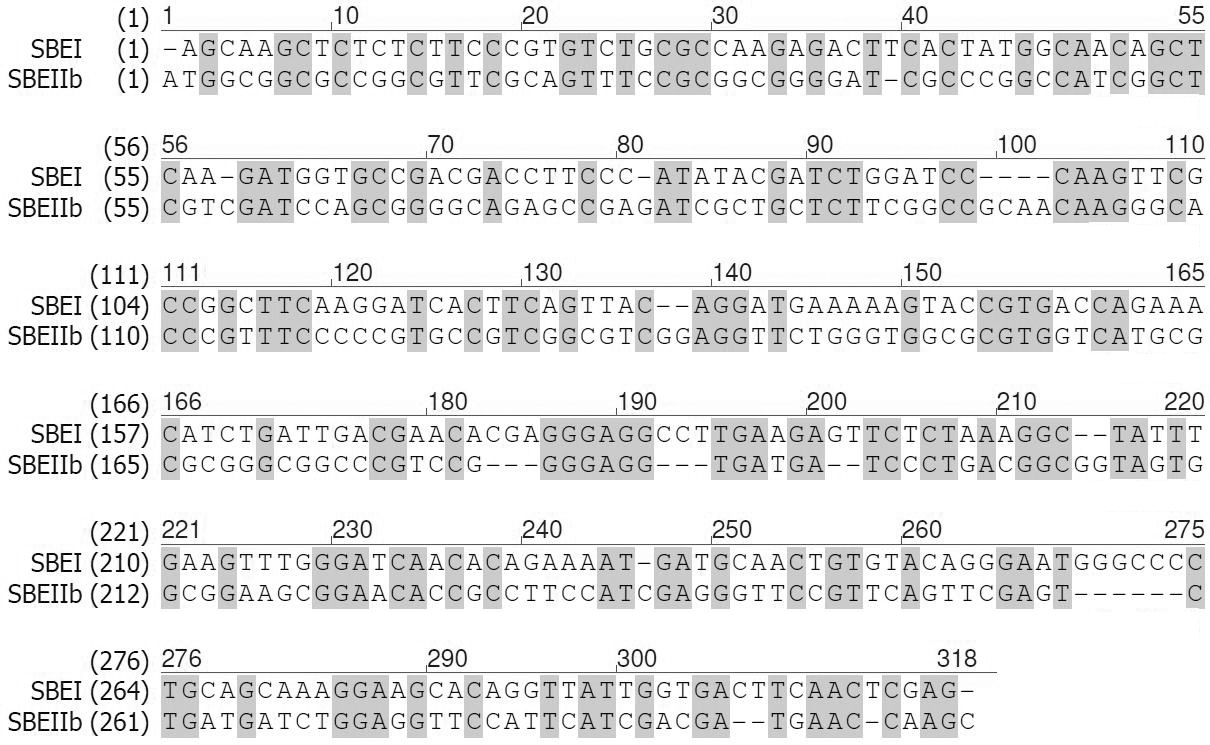


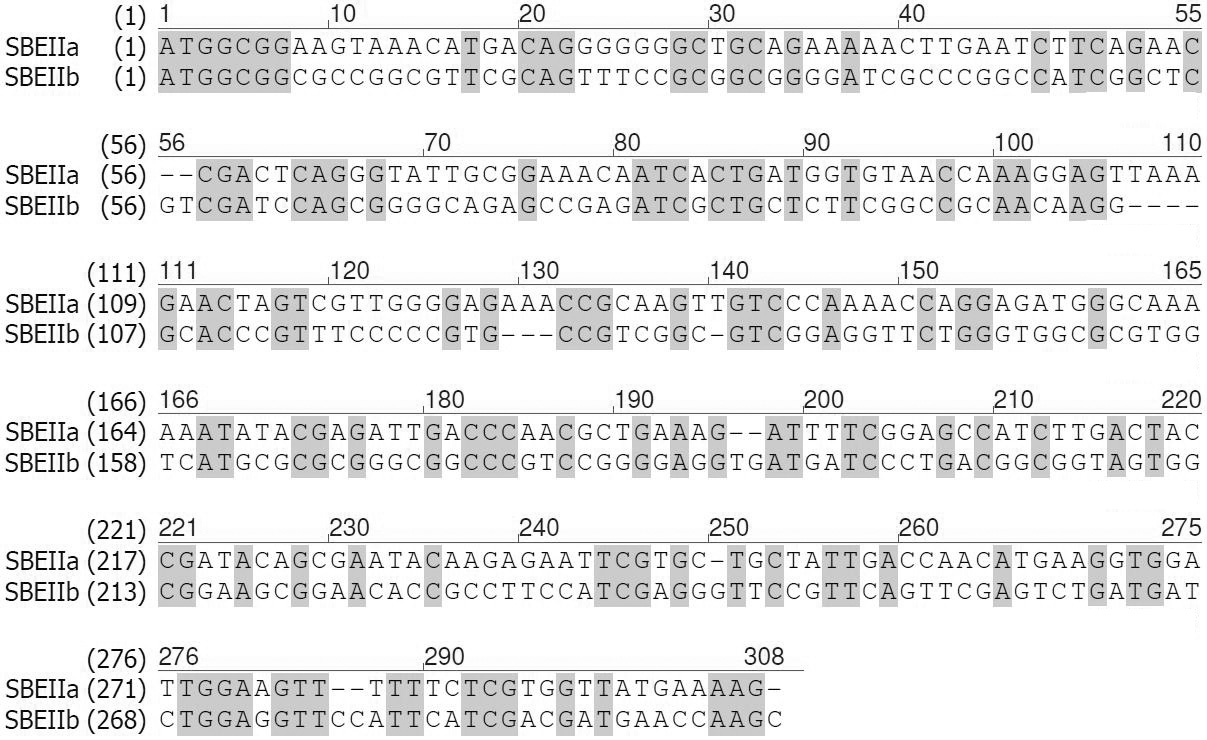

Supplement: Additional file 1 — SBE target sequences. Target sequence of SBEI, SBEIIa and SBEIIb for the chimeric SBE RNAi construct. [file 1471-2229-12-223-S1.doc]
