# Supplementary figures and images for: Concerted suppression of all starch branching enzyme genes in barley produces amylose-only starch granules
Source: BMC Plant Biol. 2012 Nov 21;12:223. doi: 10.1186/1471-2229-12-223 (PMC3537698; doi:10.1186/1471-2229-12-223)

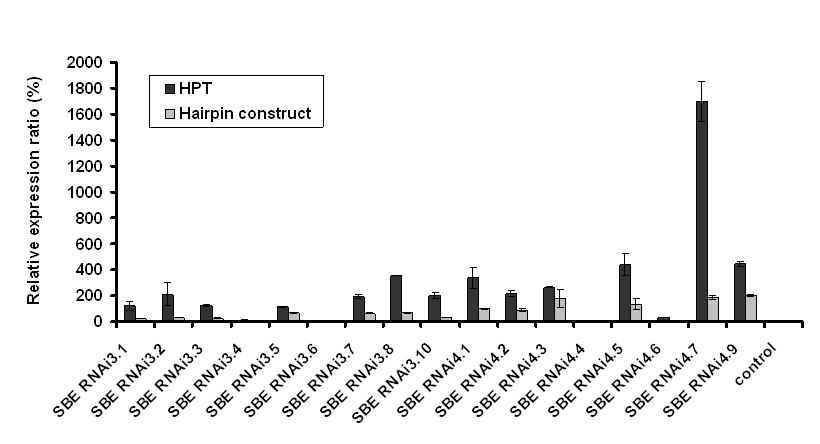

Supplement: Additional file 2 — Transgene expression analysis. Relative expression levels of selection marker gene (Hpt) and transgenic hairpin construct analysed by RT qPCR in leaves of control and transgenic T1 lines (three technical replicates each). SE is indicated. [file 1471-2229-12-223-S2.doc]

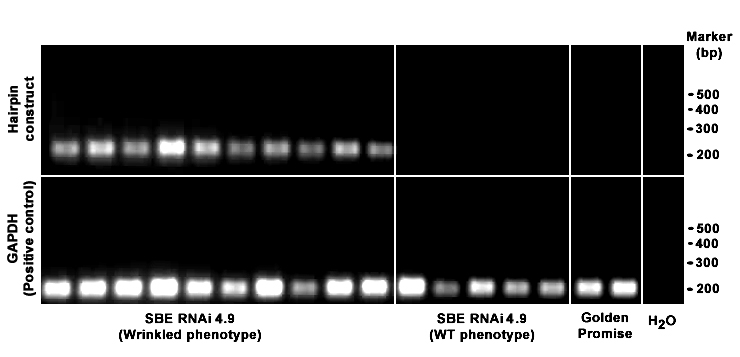

Supplement: Additional file 4 — PCR of SBE RNAi 4.9 T1genomic DNA. A PCR was performed using the primers Hairpin Fw and Rev (Figure 1a) to detect presence of the transgene hairpin in the genomic DNA of SBE RNAi 4.9 T1 grains. Primers for GAPDH was used as a positive control. The experiment was conducted in triplicate showing the same result. [file 1471-2229-12-223-S4.docx]

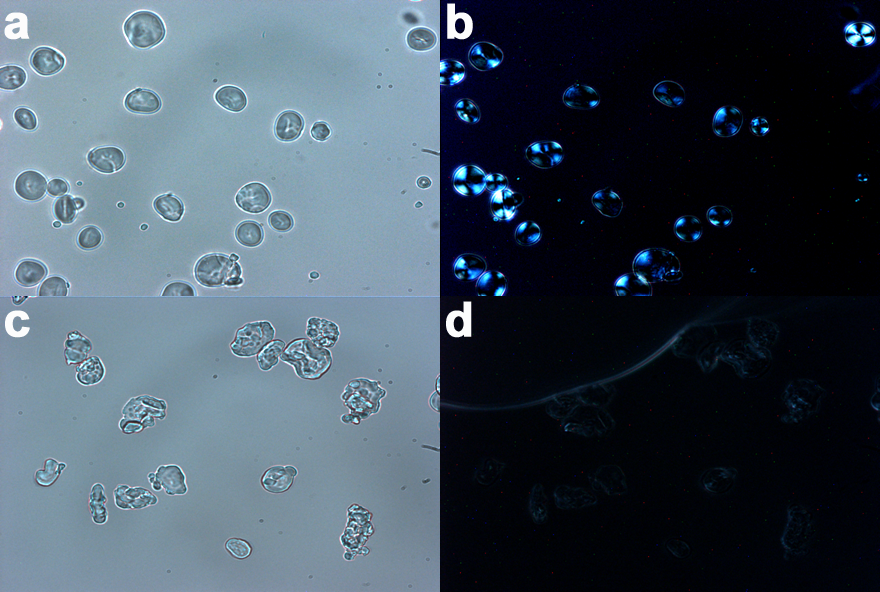

Supplement: Additional file 6 — Polarization microscopy. Bright field microscopy image (left) and polarized microscopy image (right) of control starch granules (a, b) and amylose-only granules from line SBE RNAi4.1 (c, d). [file 1471-2229-12-223-S6.doc]
