# Supplementary material for: Concerted suppression of all starch branching enzyme genes in barley produces amylose-only starch granules
Source: BMC Plant Biol. 2012 Nov 21;12:223. doi: 10.1186/1471-2229-12-223 (PMC3537698; doi:10.1186/1471-2229-12-223)
Supplement: Additional file 3 — Differential scanning calorimetry table. Differential scanning calorimetry (DSC) in aqueous suspension of starch extracted from control and from SBE RNAi4.1 lines. ΔH: Change in enthalpy due to starch thermal dissolution in water. [file 1471-2229-12-223-S3.doc]

| **Sample** | **Peak temperature (°C)**  **± SD** | **ΔH (J/g)** |
| --- | --- | --- |
| Control | 66 ± 0.4 | 6.8 ± 0.2 |
| SBE RNAi4.1 | Not detected | Not detected |
