# Supplementary material for: Concerted suppression of all starch branching enzyme genes in barley produces amylose-only starch granules
Source: BMC Plant Biol. 2012 Nov 21;12:223. doi: 10.1186/1471-2229-12-223 (PMC3537698; doi:10.1186/1471-2229-12-223)
Supplement: Additional file 5 — β-Glucan content. β-Glucan content in SBE RNAi4.1, SBE RNAi4.9 and control T2grains. β-glucan content is reported in percent dry weight (average values of two biological and six technical replicates). [file 1471-2229-12-223-S5.doc]

| **Sample** | **β-Glucan content (%) ± SD** |
| --- | --- |
| SBE RNAi4.1 | 3.8 ± 0.1 |
| SBE RNAi4.9 | 4.1 ± 0.1 |
| Control | 3.1 ± 0.1 |
