# Supplementary material for: Concerted suppression of all starch branching enzyme genes in barley produces amylose-only starch granules
Source: BMC Plant Biol. 2012 Nov 21;12:223. doi: 10.1186/1471-2229-12-223 (PMC3537698; doi:10.1186/1471-2229-12-223)
Supplement: Additional file 7 — Yield. (a) Average number of spikes per plant calculated in all plants from a semi-field trial (sample population: 50 plants each line). (b) Average number of grains per spike in 25 plants per line from a semi-field trial. (c) Average single grain mass calculated for 25 plants per line from a semi-field trial. (d) Average yield per plant in milligram of grain produced calculated in a sample of 25 plants per line from semi-field trial. (e) Correlation coefficients (r) and significance level as P values for the three yield components: average yield per plant, average number of spikes per plant and average grain mass. [file 1471-2229-12-223-S7.doc]

| **Line** | **Average spikes number per plant ± SD** | **Pr > F** |
| --- | --- | --- |
| Control | 7.7 ± 2.2 | 0.0002 |
| SBE RNAi 4.1 | 6.0 ± 2.0 |

**(a)**

| **Line** | **Average number of grains per spike ± SD** | **Pr > F** |
| --- | --- | --- |
| Control | 18.6 **±** 1.7 | 0.27 |
| SBE RNAi 4.1 | 18.1 **±** 1.3 |

**(b)**

| **Line** | **Average grain mass (mg) ± SD** | **Pr > F** |
| --- | --- | --- |
| Control | 32.6 **±** 4.7 | 0.005 |
| SBE RNAi 4.1 | 29.2 **±** 3.1 |

**(c)**

| **Line** | **Average yield per plant (mg) ± SD** | **Pr > F** |
| --- | --- | --- |
| Control | 4747.8 **±** 1292.8 | < 0.0001 |
| SBE RNAi 4.1 | 3704.5 **±** 1117.6 |

**(d)**

| **Correlation coefficients (r)** | **Average yield per plant (P)** | **Average spikes number per plant (P)** | **Average grain mass (P)** |
| --- | --- | --- | --- |
| **Average yield per plant (P)** | 1 (ns) | 0.789 (<0.0001) | 0.308 (0.029) |
| **Average spikes number per plant (P)** | 0.789 (<0.0001) | 1 (ns) | -0.155 (0.287) |
| **Average grain mass (P)** | 0.308 (0.029) | -0.155 (0.287) | 1 (ns) |

**(e)**
