# Supplementary material for: Concerted suppression of all starch branching enzyme genes in barley produces amylose-only starch granules
Source: BMC Plant Biol. 2012 Nov 21;12:223. doi: 10.1186/1471-2229-12-223 (PMC3537698; doi:10.1186/1471-2229-12-223)
Supplement: Additional file 8 — Germination frequency. Germination frequency in soil of barley grains from SBE RNAi 4.1 and control lines. [file 1471-2229-12-223-S8.doc]

| **Line** | **Sowed Grains** | **Germinated Grains** | **Germination Ratio (%)** |
| --- | --- | --- | --- |
| SBE RNAi4.1 | 60 | 59 | 98 |
| Control | 60 | 59 | 98 |
