# Supplementary material for: Concerted suppression of all starch branching enzyme genes in barley produces amylose-only starch granules
Source: BMC Plant Biol. 2012 Nov 21;12:223. doi: 10.1186/1471-2229-12-223 (PMC3537698; doi:10.1186/1471-2229-12-223)
Supplement: Additional file 9 — Primers. Primers for RT qPCR. [file 1471-2229-12-223-S9.doc]

| Primer Name | Sequence |
| --- | --- |
| *Hpt* Fw | 5'-CGTCTGTCGAGAAGTTTCTG-3' |
| *Hpt* Rev | 5'-TCCCCAATGTCAAGCACTTC-3' |
| *Hairpin* Fw | 5´-GAGCCATCTTGACTACCGAT-3´ |
| *Hairpin* Rev | 5´-TTGTTGCGGCCGAAGAGC-3´ |
| *Sbe* I Fw | 5´-TGATTGACGAACACGAGGGA-3´ |
| *Sbe* I Rev | 5´-TCCCGTTGACATGGGAAATC-3´ |
| *Sbe* IIa Fw | 5´-GAGCCATCTTGACTACCGAT-3´ |
| *Sbe* IIa Rev | 5´-GGGAGGAAAATCTCCCAAAC-3´ |
| *Sbe* IIb Fw | 5´-AAGCGGAACACCGCCTTC-3´ |
| *Sbe* IIb Rev | 5´-GGTTGTGGCACAATGCGTAT-3´ |
| *SSI Fw* | 5´-TCGAAGGGATTGCTGAGGAT-3´ |
| *SSI Rev* | 5´-AGCAGCAAGAGCAATTGGCA-3´ |
| *SSIIa* Fw | 5´-CCGCTGTACAAGAAGACCTT-3´ |
| *SSIIa* Rev | 5´-CAAGACCACCTGTTTTGCAC-3´ |
| *SSIIIa* Fw | 5´-TGAAAAGAAAGGCTGAGAGAAG-3´ |
| *SSIIIa* Rev | 5´-AGGAGCATCTAAACCAACCC-3´ |
| *SSIV* Fw | 5´-CTGCATTTGTTGCACCTCTTTA-3´ |
| *SSIV* Rev | 5´-CAACTGCACCCTTAACAGCA-3´ |
| *GBSSIa* Fw | 5´-TCATCTCCGAGATCAAGGTC-3´ |
| *GBSSIa* Rev | 5´-GAGGTTGAGGATCCTGGG-3´ |
| *GBSSIb* Fw | 5´-CGGCACAGGGAAGAAGAAAA-3´ |
| *GBSSIb* Rev | 5´-GATGGGACCAACTCCATAGC-3´ |
| *GWDI* Fw | 5´-AATTCGTGGTGGATCAGCTG-3´ |
| *GWDI* Rev | 5´-CACCTTGCAATTCCTTGCTC-3´ |
| *GAPDH Fw* | 5´-GCTCAAGGGTATCATGGGTTACG-3´ |
| *GAPDH Rev* | 5´-GCAATTCCACCCTTAGCATCAAAG-3´ |
